# A cytogenetic study of couples with recurrent spontaneous abortions and infertile patients with recurrent IVF/ICSI failure

**DOI:** 10.4103/0971-6866.42319

**Published:** 2008

**Authors:** Hossein Mozdarani, Anahita Mohseni Meybodi, Shabnam Zari-Moradi

**Affiliations:** 1 Department of Medical Genetics, School of Medical Sciences, Tarbiat Modares University, Tehran, Iran; 2 Department of Infertility Genetics, Royan Institute, Tehran, Iran

**Keywords:** Abnormal karyotypes, infertile couples, recurrent abortions, recurrent IVF/ICSI failure

## Abstract

**PURPOSE:**

This study was conducted to determine the frequency and contribution of chromosomal abnormalities in miscarriages and in couples with recurrent *in vitro* fertilization/intra cytoplasmic sperm injection (IVF/ICSI) failure.

**MATERIALS and METHODS:**

A total of 221 individuals; 79 with three or more recurrent spontaneous abortions and 142 with at least three IVF/ICSI failures. Chromosomal analysis from peripheral blood lymphocytes was performed according to standard cytogenetic methods using G-banding technique.

**RESULTS:**

Abnormal karyotype was found in 21 (9.50%) individuals. Of these 21 subjects, 4 (19.04%) exhibited sex chromosomal abnormalities and 17 (80.96%) had autosomal abnormalities. Male partners had significantly higher chromosomal abnormalities (5.88%) than of females (3.61%). These abnormalities were also higher in patients with recurrent spontaneous abortions than with IVF/ICSI failure (*P* < 0.05).

**CONCLUSIONS:**

These data may be indicative that chromosomal abnormalities are involved more in spontaneous abortions than in recurrent IVF/ICSI failure. Cytogenetic analysis could be valuable for these couples when clinical data fail to clarify the cause.

## Introduction

Reproduction in human being is genetically risky process and terribly incompetent. The most common outcome of conception is embryonic or fetal death. Near one-third of conceptions do not result in the delivery of a baby. Miscarriages are clinically detectable pregnancies that fail to progress. They are common and often remain unexplained, although it has shown that a major cause of this demise is attributed to chromosomal abnormalities. It is demonstrated by the high frequency of abnormalities found in sample fetal or embryonic tissue.[1] When a woman has had two or more miscarriages, she will be under the care of gynecologist to seek professional help in the hope that a cause and care will be found.[2] In the other way, the development of IVF and ICSI and related techniques has increased the possibility of obtaining babies from infertile patients. Today recurrent implantation failure is the major reason for women completing several IVF/ICSI attempts without having achieved a child, and is probably also the explanation for many cases of unexplained infertility. Most causes of recurrent miscarriage are still poorly elucidated, but from a theoretical point of view recurrent implantation failure and recurrent miscarriage are suggested to have partly overlapping causes.[3] It is well known that lower implantation rate and higher spontaneous abortions rate are closely related with the chromosomal abnormalities of both parents. Even in some patients, unexplained multiple IVF/ICSI failure has been frequently reported.[4] An association between human infertility and chromosomal abnormalities has been known for long time;[5] thus, it would not be unusual to find chromosomal abnormalities in couples attending an infertility clinic.

Studies of Gianaroli *et al.* showed that infertile patients with poor prognosis have an increased risk of having embryos with chromosomal abnormality, which could be one of the main reasons of implantation failure or recurrent spontaneous abortions.[6] With the development of Assisted Reproduction Technologies (ART), genetic counseling and screening of couples take a greater importance. As a result, karyotyping of White Blood Cells of every person attending the infertility clinic would be necessary to identify those with genetic defects. The aim of this study was to examine two groups of couples from infertile marriage, in an attempt to identify any clinical abnormality that could be of predictive value for chromosomal aberrations.

## Materials and Methods

The patient population consisted of 221 Iranian individuals (102 men and 119 women) who attended the infertility clinic with a history of repeated spontaneous abortion and IVF/ICSI failure. We divided our samples in two groups: first group consisted of couples with recurrent spontaneous abortion (79 individuals) and the second group was those who had at least three IVF or ICSI failures (142 individuals). On epidemiological evidence, the definition of recurrent miscarriage should be three or more consecutive pregnancy losses. Women meeting the definition can be subdivided into primary and secondary groups, respectively, consisting of those who have lost all previous pregnancies and those who have had one successful pregnancy followed by consecutive losses.[7] In the present study, the patients had a history of three or more abortions and did not have any children. Their managements were started with clinical examination by gynecologist and urologist and then by a genetic counselor. The anatomical problems were ruled out by gynecologist and urologists. In all women antibodies against toxoplasmosis, rubella and cytomegalovirus (CMV) were analyzed by enzyme-linked immunosorbent assay (ELISA) Trinitin 99% kits. The blood samples of both male and female partners were subjected to a white cell chromosome analysis. After genetic counseling, family pedigree was drawn by genetic counselor. Karyotyping was conducted by analysis of G-banded chromosomes using 5 mL heparinized peripheral blood sample. Metaphase spreads were made from phytohemaglutinin stimulated peripheral lymphocytes using standard cytogenetic techniques. Cultures were harvested and Karyotyping was performed on G-bands produced with trypsin and Giemsa (GTG)-banded chromosome preparations. The chromosomal status was analyzed using CytoVision Ultra ver.4.0 from Applied Imaging (New Castle, UK). At least 25 metaphases were analyzed for each patient. If there was any sign of mosaicism, 50 metaphases from two independent cell cultures were analyzed. All chromosomal abnormalities were reported in accordance with the current international standard nomenclature.[8] The X^2^-test and one way analysis of variance (ANOVA) were used for statistical evaluation by SPSS software (version 11.5). The level of *P* < 0.05 was considered as significance.

## Results

**Figure 1:**
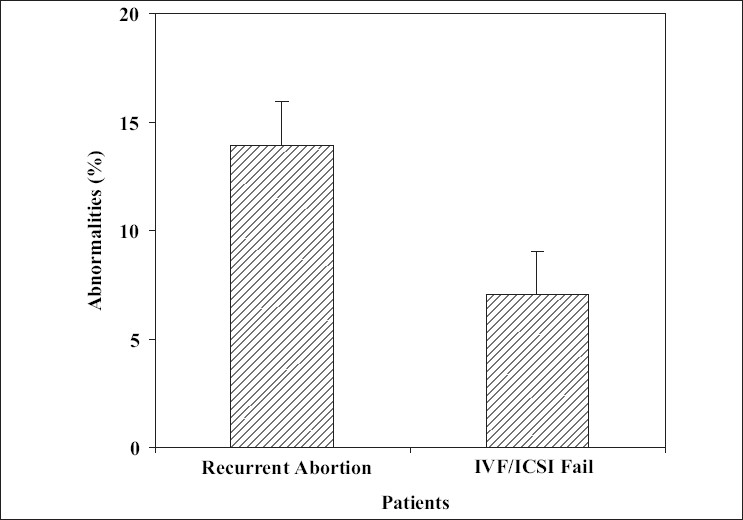
The abnormality rate in different study groups

**Figure 2:**
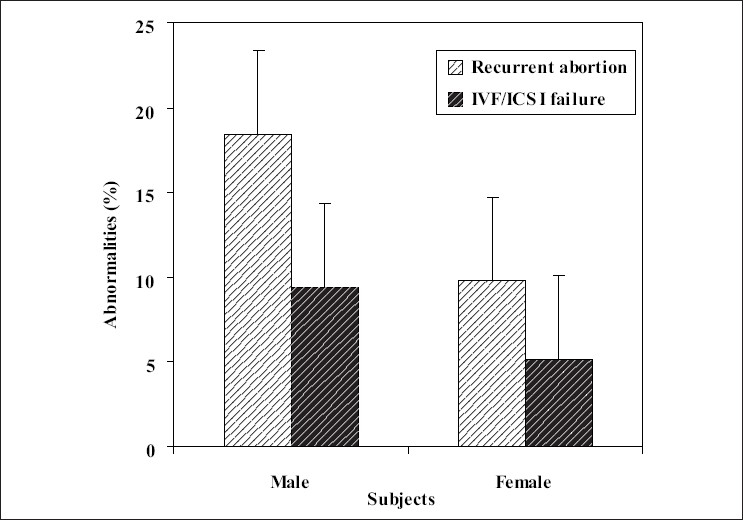
The percentage of abnormal male and female karyotype in different study groups

The median age of male partners was 35.36 ± 0.85 and the median age of female partners was 31.0 ± 1.1. There did not appear to be an age-related distribution of gonosomal aberrations. The results obtained are summarized in Tables 1-3 and shown in Figures 1 and 2.

**Table 1:** Frequency and types of autosomal chromosome aberrations in male and female partners

Male autosomes			Female autosomes		
	
Aberrations	Karyotype	Frequency	Aberrations	Karyotype	Frequency
Numerical	-	-	Numerical	46XX/47XX + 21	1
Marker chromosome	47XY + Marker	1	Marker chromosome	-	-
Translocations	45XY; t(14;15)(q10;q10)	1	Translocations	45XX; t(13;14)(q10;q10)	1
	46XY; der (2;8)(p13;p23)	1			
Minor structural	46XY, 22 S^++^	2	Minor	46XX, 22 S^++^	1
abnormalities	46XY, 21 S^++^	1	structural	46XX, 14 S^++^	1
	46XY, 16 qh^++^	1	abnormalities	46XX, 9 qh^++^	1
Inversions	46XY; Inv (9)(p11;q12)	5	Inversions	-	-

**Table 2:** Frequency and types of sex chromosome aberrations in male and female partners

Male sex chromosomes			Female sex chromosomes		
	
Aberrations	Karyotype	Number	Aberrations	Karyotype	Number
Complete numerical	47XXY	1	Complete numerical	47XXX	1
Mosaicism	-	-	Mosaicism	46XX/47XXX	1
				46XX/45X	1

**Table 3:** Frequency of cytogenetically normal and abnormal individuals in studied subjects

Patients	Normal			Abnormal			Total number of individuals
			
	Male	Female	Total	Male	Female	Total	
Recurrent Abortions	31	37	68	7	4	11	79
IVF/ICSI failure	58	74	132	6	4	10	142

Abnormal karyotypes were found in 21 (9.50%) of the 221 subjects studied. We were not aware of any data available on the rate of anomalies in an equivalent fertile population. Of these 21 subjects, 4 (19.04%) exhibited sex chromosomal abnormalities. The remaining 17 (80.96%) had autosomal abnormalities. Of these four individuals with sex chromosomal abnormalities, one was found to have Kleinfelter's syndrome, which constituted 4.76% of the total group studied. One of the patients had super female syndrome and the rest of them had mosaicism of X chromosome.

Of those 17 individuals exhibiting autosomal anomalies, five showed inversion, one had marker chromosome, one with the mosaicism of chromosome 21, three had autosomal translocation, and the seven remaining showed a variety of minor autosomal abnormalities [Tables 1 and 2].

Inversion of chromosome 9, normally not considered as a pathological chromosome alteration, was also included in Table 1 for the sake of completeness. The frequency of aberrations was 5.88% in the male partners. Numerical or structural abnormalities were documented in 3.61% of the female partners. There was significant difference for the rate of aberrations between male and female partners (*P* < 0.05) [Figure 2].

The results also were analyzed according to the group of patients and are shown in Table 3 and Figures 1 and 2. The rate of anomalies was higher in both male and female with recurrent abortions history than those who had IVF/ICSI failure (*P* < 0.05). Statistical analysis of data showed a statistically significant difference for the rate of abnormalities between the two groups under investigation.

## Discussion

Most of the spontaneous miscarriages are caused by chromosomal abnormalities in the embryo or fetus.[9–11] The genetic factors represent more than 50% of early gestation spontaneous abortion and associated with fetal chromosomal abnormalities.[12] The genetic etiology for multiple spontaneous pregnancy loss includes an unbalanced chromosome rearrangement, which may be the result of one parent being a carrier for a balanced chromosome rearrangement.[13] In 4-8% of couples with recurrent pregnancy loss, at least one of the partners has chromosomal abnormality that probably contains balance chromosomal abnormalities.[2] The results of the present study showed 13.92% chromosomal abnormalities in these individuals [Figure 1]. The prevalence of chromosomal aberrations among couples with repeated spontaneous abortions varied in different studies, from none[14] to as high as 21.4%.[15] In the present study, incidence of chromosomal abnormalities among studied couples with repeated abortions was 13.92% which is higher than chromosomal aberrations prevalence among couples with repeated abortions found by Pescia *et al.* (5%),[16] Palanduz *et al.* (6.5%),[17] Al Hussain *et al.* (7.7%),[18] and Mohammed (7.8%),[19] but close to that reported by Butler and Hamill (17.8%).[20] On the other hand, the types of abnormalities can play an important role in the effect of aberration. Fryns and Van Buggenhout reported that of the chromosome abnormalities observed in couples with repeated abortions, two-thirds were balanced autosomal translocations.[21] The rate of these abnormalities in this research was less than their report and was 14.28% of all the aberrations. X-chromosome mosaicism, which was seen in our karyotypes, is usually associated with abnormal development and reproductive performance, including recurrent spontaneous abortion.[22] We demonstrated X-chromosome mosaicism in two females (1.68%) out of 119 women experiencing recurrent abortion and IVF/ICSI failure. However, the reproductive performance of X-chromosome mosaicism is highly variable and difficult to define.[23] The incidence of karyotype abnormalities among infertile men has been reported to range between 2.2% and 19.6%[24–26] and numerical or structural abnormalities have been documented in near 10% of the female partners[27] similar to our observations shown in Figure 2. Moreover, other studies showed that structural chromosomal abnormality is the most common chromosomal abnormality in couples with recurrent abortions especially couples undergoes ART.[28,29] *In vitro* fertilization plus preimplantation genetic diagnosis (PGD) is an important step in the management of these couples.[10] One in 500 people carries a balanced translocation. When one member of a couple carries a balanced chromosome translocation, the risk of having a miscarriage is approximately doubled. In 3-5% of couples with recurrent miscarriage, one partner has a balanced translocation.[2] According to some researches, early on, genetically normal and abnormal embryos have similar appearance. On the basis of morphology alone, the genetic integrity cannot be determined; therefore, transferring of embryo in IVF and ICSI is some how risky and nonselective, so the risk of transferring the abnormal embryo is still persist. It can be resulted in IVF or ICSI fail.[30,31] The commonly reported human inversion was inversion (9) (p12;q13). The role of pericentric inversion of chromosome 9 in infertility and pregnancy losses is still very controversial.[32] Although inversion (9) has been associated with repeated spontaneous abortions in several families, studies on unselected series of couples have usually failed to demonstrate a relationship between inversion (9) and repeated abortions.[33,34] This study has shown that the incidence and distribution of total chromosomal abnormalities among our patients are comparable to that reported worldwide. In the future to complete this study, cytogenetic analysis of the abort uses should be done, which help the family in other pregnancies. In other research has indeed documented that both syndromes can be caused by the same embryonic chromosomal abnormalities and the same maternal endocrine, thrombophilic, and immunological disturbances. Consequently, many treatments attempting to normalize these abnormalities have been tested or are currently used in women with both recurrent implantation failure and recurrent miscarriage. However, no treatment for the two syndromes is at the moment sufficiently documented to justify its routine use.[3] We concluded from all the previous results that cytogenetic studies should be performed to all couples with two or more spontaneous abortions and also in patients with recurrent IVF/ICSI failure. In a case of detected chromosomal aberration; the patient should be counseled individually according to the type of anomaly. This study should help physicians working in the region to realize the contribution of chromosomal abnormalities to cases of repeated fetal loss. It should also help in setting priorities of cytogenetic screening in individual cases. With subsequent possibility of the performance of PGD using the routine *in vitro* fertilization, biopsy of the embryos allows the selected transfer of chromosomally balanced embryos. The translocation PGD has been applied successfully in several centers and should be now considered as a realistic treatment option for translocation carrier.
